# Dendrimers: Exploring Their Wide Structural Variety and Applications

**DOI:** 10.3390/polym15224369

**Published:** 2023-11-09

**Authors:** María Pérez-Ferreiro, Adrián M. Abelairas, Alejandro Criado, I. Jénnifer Gómez, Jesús Mosquera

**Affiliations:** CICA—Centro Interdisciplinar de Química e Bioloxía, Universidade da Coruña, Rúa as Carballeiras, 15071 A Coruña, Spain

**Keywords:** traditional dendrimers, Janus dendrimers, supramolecular dendrimers, shape-persistent dendrimers, rotaxane dendrimers

## Abstract

Dendrimers constitute a distinctive category of synthetic materials that bear resemblance to proteins in various aspects, such as discrete structural organization, globular morphology, and nanoscale dimensions. Remarkably, these attributes coexist with the capacity for facile large-scale production. Due to these advantages, the realm of dendrimers has undergone substantial advancement since their inception in the 1980s. Numerous reviews have been dedicated to elucidating this subject comprehensively, delving into the properties and applications of quintessential dendrimer varieties like PAMAM, PPI, and others. Nevertheless, the contemporary landscape of dendrimers transcends these early paradigms, witnessing the emergence of a diverse array of novel dendritic architectures in recent years. In this review, we aim to present a comprehensive panorama of the expansive domain of dendrimers. As such, our focus lies in discussing the key attributes and applications of the predominant types of dendrimers existing today. We will commence with the conventional variants and progressively delve into the more pioneering ones, including Janus, supramolecular, shape-persistent, and rotaxane dendrimers.

## 1. Introduction

Dendrimers are hyper-branched macromolecules characterized by large numbers of end-group functionalities and a compact molecular structure. They consist of a central core molecule where multiple branches emerge, giving rise to a hierarchical and well-defined architecture. The branches consist of repeated units or monomers that are chemically linked together in a controlled manner. Since their introduction in the 80s [1], this revolutionary class of materials has played a pivotal role in pushing the boundaries of polymer chemistry, effectively bridging the gap between synthetic and biological polymers. Remarkably, dendrimers exhibit a plethora of exceptional properties that bear striking resemblance to those of biological macromolecules, notably proteins. These properties encompass the following:

(i) **Monodispersity**: Dendrimers can be synthesized as monodispersed materials, resulting in a uniform and well-defined molecular weight distribution. Dendrimers’ monodispersity contributes to their functionality and reliability in various applications.
(ii) **Nanometer size**: The size of dendrimers depends on their generation (vide infra), but in general, they are several nanometers big, i.e., very similar to proteins. This is a relevant advantage with respect to molecules since a large surface can be applied to performing multivalency interactions that determine their biological behavior.
(iii) **Globular shape**: Dendrimers possess a characteristic globular shape, which arises from their highly branched and symmetric structure. This globular architecture is crucial to their interactions with other molecules.
(iv) **Adaptable surface**: Dendrimers offer a customizable platform for the attachment of an extensive array of molecules. This process of functionalization empowers precise modulation of their physicochemical attributes and governs molecular interactions transpiring upon their surfaces.
(v) **Presence of cavities**: The globular shape of dendrimers creates internal cavities or void spaces within their structure. These cavities can accommodate guest molecules, drugs, or other functional moieties, providing a controlled and protected environment.

The remarkable properties of dendrimers, reminiscent of biological polymers, have opened up vast possibilities in diverse fields, including nanotechnology, medicine, catalysis, and materials science. Recent years have witnessed the publication of a few reviews that have extensively explored the properties and applications of the most traditional dendrimers [2,3,4,5], i.e., polyamidoamine (PAMAM), poly(propylene imine) (PPI), polylysine (PLL), and polyester dendrimers. In contrast, our review aims to distinguish itself by shedding light on the emerging and innovative variants of dendrimers. Specifically, our study is dedicated to elucidating the characteristics and potential applications of novel dendrimer types. By expanding the scope of information, we seek to contribute to a comprehensive understanding of the diverse and evolving landscape of dendrimer research.

## 2. Dendrimer Structure

The physicochemical properties of dendrimers are determined by the three constituent parts: the core, the branches, and the end-group functionalities. While exceptions exist, it is generally observed that the core has a relatively lower impact on properties such as solubility, polarity, or the ability of the dendrimer to interact with molecules. However, besides its structural role, the core molecule can endow the dendrimer with additional capabilities. For example, porphyrins have been applied as dendrimer cores, resulting in dendrimers with fluorescence emission and photosensitizing features [6]. Another example is the utilization of cyclophane as the core, which allows the dendrimer to encapsulate specific molecules [7]. 

On the contrary, the chemical nature of the branches significantly influences the following dendrimer properties:

(i) **Synthesis**: Different types of branches, such as amine-, ester-, or ether-based branches, require specific synthetic methodologies to achieve the desired dendrimer architecture. Careful selection of branching units allows for precise control over dendrimer growth, size, generation, and molecular structure.
(ii) **Flexibility**: Branching units greatly affect the rigidity and flexibility of dendrimers. Rigid branches, like aromatic or bulky groups, lead to more rigid dendrimers, while flexible or aliphatic branches introduce greater flexibility [7].
(iii) **Porosity**: The properties of the branches impact the accessibility of molecules to the dendrimer’s interior. Branches with low solubility in a solvent can cause dendrimers to contract and restrict the solvent’s accessibility to the dendrimer’s interior, also affecting the capabilities of the dendrimer to encapsulate molecules.
(iv) **Stability**: Some branches have chemical groups that can be unstable in certain conditions, for example, under heating. Other ones, such as ester or amide bonds, can be broken by enzymes, making the dendrimer biodegradable.

Finally, the end-group functionalities are the ones located at the outermost ends of the dendrimer branches, and determine the surface properties, reactivity, and interactions of the dendrimers with other molecules. They significantly influence the overall behavior of dendrimers, including control over solubility. By carefully selecting the appropriate end-groups, dendrimers can be tailored to exhibit water solubility or solubility in organic solvents [8]. The remarkable chemical diversity of end-group functionalities allows virtually any molecule to be attached as a terminal group, offering limitless possibilities for customizing dendrimers with specific properties and functionalities. Importantly, the number of end-group functionalities and the dendrimer size is mainly determined by the generation of the dendrimer, i.e., the number of repeated branching cycles that are performed during its synthesis. Each generation adds a layer of branches, increasing the overall size and the number of end-group functionalities.

**Figure 1:**
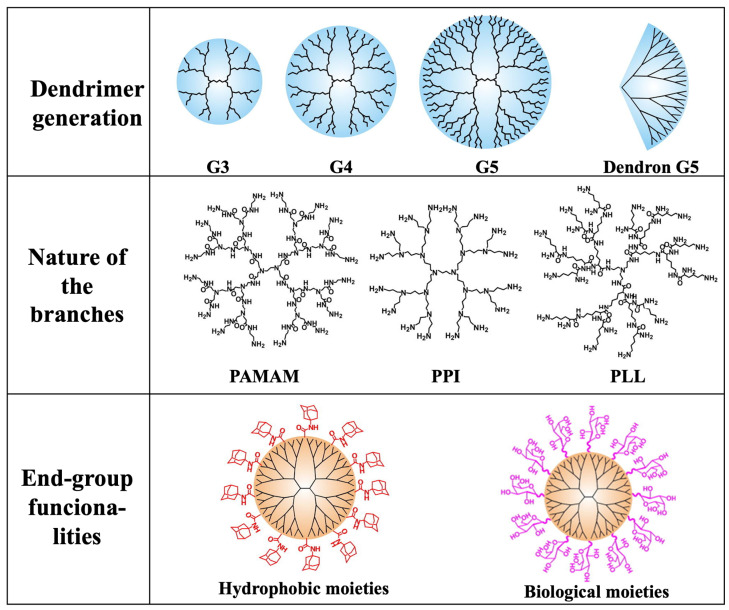
Schematic representation of the main parameters that determine the properties of a dendrimer. (Adapted from Ref. [9]).

In conclusion, the properties of the dendrimers are mainly determined by (i) the generation (G1, G2, G3, …), (ii) the chemical nature of the branches, e.g., PAMAM, PPI, etc., and (iii) the end-group functionalities (Figure 1). 

## 3. Synthesis of Dendrimers

Two primary approaches for dendrimer formation have been defined: convergent and divergent [10]. Each of these approaches carries unique advantages and notable distinctions. The meticulous selection of a suitable strategy stands as a pivotal factor, as it will define the distinctive characteristics of the macromolecule in each scenario. 

### 3.1. Divergent Approach

This methodology reported by Tomalia et al. [1] is based on the stepwise growth of dendrimers, starting from a core containing diverse binding groups. Sequential reactions are then performed to expand the dendrimer over several generations, depending on the specific sequences applied.

**Figure 2:**
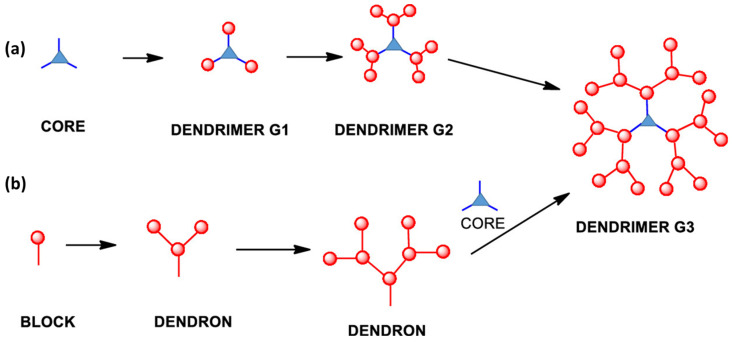
Cartoon representation of (**a**) divergent and (**b**) convergent synthesis. Reprinted with permission from ref [11]. Copyright 2019 American Chemical Society.

These reactions can involve various functional groups, such as amines, boronic acids, alcohols, among others, but they all follow a similar procedure (Figure 2a). The process initiates with a reaction at the core, employing non-reactive functional groups that appear at the periphery of the newly formed macromolecule. Subsequently, a deprotection or activation step is undertaken to convert these peripheral groups into reactive entities, which facilitates the subsequent reaction. This iterative process is repeated for subsequent generations until the final dendrimer is achieved. 

The main advantage of this methodology lies in its capability to generate vast and complex structures via the exponential growth of the dendrimer in each generation. This property also aids in the purification process, as the substantial difference in size between the dendrimer and monomers facilitates their separation. However, a significant challenge arises from the possibility of side reactions occurring in some dendrimers at each step. This is attributed to the increasing number of reactive groups with each successive generation. Consequently, the final yield of each generation is diminished, leading to the formation of dendrimers with structural defects due to the presence of by-products [12].

To counteract the exponential increase in reactive groups and to minimize the formation of by-products, the use of a substantial excess of monomers in each step of the approach is necessary. This excess ensures that the desired reactions are favored and helps reduce unwanted side reactions.

### 3.2. Convergent Approach

This methodology, initially reported by Hawker and Fréchet in 1990 [13], involves the stepwise formation of different branches, known as dendrons, which ultimately culminates in the synthesis of the dendrimer’s final macrostructure via a final bond with the core.

The synthesis of these dendrons follows a process like the divergent approach (Figure 2b). The primary advantage of this approach is the improved control over the dendrimer’s structure, thanks to the smaller size of the dendrons compared to the dendrimer itself. This necessitates fewer equivalents of monomer in each synthesis step and a reduced formation of by-products during the reactions can be observed. Enhanced control also enables the precise introduction of the desired functional groups at the dendrimer’s periphery, which significantly impacts the final properties of the macromolecule [14]. 

However, a notable drawback is the steric hindrance encountered during the final coupling of the dendrons with the core. The large size of the dendrons impedes the reaction of other branched arms, limiting the formation of dendrimers with fewer generations compared to the divergent approach. Despite this limitation, the convergent approach remains a valuable strategy for constructing dendrimers with controlled structures and desirable functionalities. It offers significant advantages in specific applications where meticulous control and minimized by-products stand as crucial considerations.

## 4. Traditional Dendrimers

Apart from the generation of the dendrimer, their most common classification is based on the chemical nature of their branches. Herein, we defined traditional dendrimers as symmetric macromolecules formed by a single type of flexible branches. Under this definition, the most common dendrimers applied nowadays are included, for which a brief summary of the main properties is included below.

### 4.1. PAMAM Dendrimers

These are comprised of repeating units of methyl acrylate and ethylenediamine; therefore, they contain amine and amide groups in their interior. PAMAM was the first dendrimer synthesized and commercialized. These dendrimers usually feature terminal amino groups, which confer a cationic nature and remarkable hydrophilicity. Additionally, those groups can be straightforwardly functionalized to modulate their physicochemical properties [15]. PAMAM finds its primary utility within the realm of biomedical applications such as a gene delivery unit, an oral drug delivery vehicle, or even an activator of the immune system [16]. It is worth mentioning that the stability of PAMAM dendrimers is relatively lower in comparison with other dendrimers. This is attributed to their susceptibility to retro-Michael reactions when exposed to high temperatures, which imposes restrictions on their potential applications. [17] PAMAM synthesis consists of performing a Michael addition using a large excess of methyl acrylate, and subsequently, an amidation reaction with ethylenediamine.

### 4.2. PPI Dendrimers

PPI dendrimers are a distinct class of dendrimers that consists of repeating propyleneimine units. Similar to PAMAM dendrimers, these dendrimers possess remarkable hydrophilicity with excellent water solubility due to their terminal cationic amino surface. However, their internal part is more hydrophobic than PAMAM as was shown by using the solvatochromic probe phenol blue [18,19]. This property of PPI dendrimers makes them highly desirable for the task of delivering drugs that have poor solubility in water [20]. Moreover, PPI dendrimers exhibit high stability and can be readily prepared with a high yield, making them commercially available. Their more efficient synthesis consists of using a Michael addition between the primary amines and acrylonitrile, and subsequently a heterogeneous hydrogenation with a Raney cobalt catalyst [21]. 

### 4.3. PLL Dendrimers

These are unique macromolecules that are constructed with repeating L-lysine units linked together using amide bonds. The presence of the asymmetric L-lysine amino acid imparts chirality on the dendrimer structure, resulting in an inherently chiral molecule. Furthermore, PLL dendrimers exhibit an asymmetrical branching pattern, where the branches possess different lengths, contributing to their complex three-dimensional architecture [22]. As in the case of PAMAM and PPI dendrimers, these dendrimers also possess amino groups as end-group functionalities, which can be utilized to enhance their solubility in aqueous as well as organic environments. Notably, an intriguing aspect of PLL dendrimers is their biodegradability, owing to their composition based on peptide bonds. This attribute renders them highly suitable for a wide range of biomedical applications, making them promising candidates in the field [23]. In fact, PLL dendrimers are currently applied in health products for the treatment and prevention of bacterial vaginosis and the prevention of sexually transmitted diseases [24]. 

### 4.4. Polyester Dendrimers

**Figure 3:**
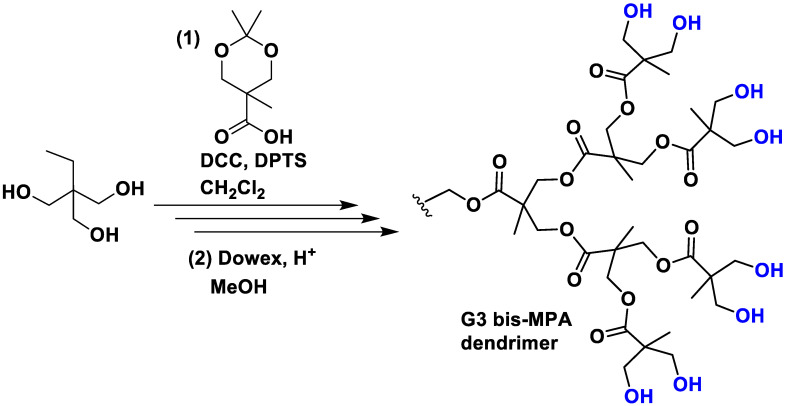
Synthesis of a G3 bis-MPA dendrimer. DCC is the coupling agent N,N′-dicyclohexylcarbodiimide and DPTS is the molecule 4-(dimethylamino)pyridinium 4-toluenesulfonate that acts as the catalyst.

These are a unique class of dendrimers in which the components are linked using ester bonds. While various types of polyester dendrimers have been reported, those based on 2,2-bis(hydroxymethyl)propanoic acid (bis-MPA) are the most prevalent. One of the notable advantages of bis-MPA is its availability at a low cost, making it an economically viable option. Its preparation consists of an esterification in dichloromethane with the DCC coupling agent in the presence of the catalyst DPTS (Figure 3). Subsequently, the protection group was removed easily under mild conditions by stirring the acetonide derivatives in MeOH in the presence of the acidic resin Dowex 50W [25]. Nowadays, several companies offer dendrons and dendrimers of bis-MPA, providing accessible options for researchers interested in utilizing these structures. Typically, the end-group functionalities of these dendrimers consist of alcohol moieties, which offer the versatility to bind other functionalities, often via the formation of carbamates [26]. Moreover, the presence of these alcohol moieties not only imparts remarkable water solubility but also offers the advantage of reduced toxicity compared to the previous dendrimers due to their neutral charge. Importantly, MPA dendrimers exhibit superior biodegradability compared to PLL polymers, primarily due to the lower stability of ester bonds in comparison to amide bonds. For this reason, they are extensively applied in biological applications [27].

### 4.5. Polyether Dendrimers

These are another type of dendrimer closely related to polyester dendrimers. The key distinction between these two families lies in their chemical bonds, as polyether dendrimers are bound using ester bonds. This difference in bonding gives polyether dendrimers a significant advantage in terms of stability compared to polyester dendrimers, particularly when it comes to chemical and biological degradation. Among the polyether dendrimers, poly(benzyl ether) dendrimers stand out as the most representative polyether dendrimer [28]. A key characteristic of this dendrimer is that their branch structure is quite hydrophobic [29].

### 4.6. Organoelement Dendrimers

Organoelement dendrimers refer to a class of dendritic macromolecules characterized by the incorporation of non-carbon elements, such as silicon [30], phosphorus [31], and boron [32], into their central core structure. Among organoelement dendrimers, those containing silicon, such as carbosilane and carbosiloxane dendrimers [30,33], are often highly representative. One key advantage of silicon-containing dendrimers is their ease of synthesis, coupled with their exceptional chemical structure, featuring robust Si–C bonds [34]. These Si–C bonds are not easily broken or affected by unwanted or side chemical reactions during their preparation, making their production simpler.

### 4.7. Applications of Traditional Dendrimers

Traditional dendrimers have found applications in a wide range of fields due to their unique properties and versatile structures [35]. However, a significant focus is placed on exploring their potential applications in the field of biomedicine, the main ones being the following:

(i) **MRI contrast agents**: Since the sensitivity of magnetic resonance imaging (MRI) to tissue type differences is relatively low, paramagnetic metals are used as contrast agents. These agents are used to shorten the relaxation times of the surrounding water protons in order to improve contrast. To minimize the need for high doses of contrast agent, dendrimers have been decorated with coordinating groups to endow affinity for metals like gadolinium. This strategy allows us to increase the efficiency of the contrast agent and also modify its biodistribution [36]. Nonetheless, metals tend to exhibit toxicity; hence, researchers are currently investigating a prospective class of contrast agents. These agents rely on dendrimers adorned with functional organic radicals, showing promising potential as MRI contrast agents [37].
(ii) **Tissue engineering**: Since dendrimers can be modified to incorporate or encapsulate a variety of biological and/or chemical substances, they have been used in tissue engineering to design artificial extracellular matrices. For instance, dendrimers can be applied to encapsulate growth factors and release the components in the native extracellular matrix in a controlled manner to enable tissue regeneration. Another option is to use dendrimers to form hydrogels that mimic natural extracellular matrices to induce the growth of the seeded cells [38].
(iii) **Gene delivery**: Dendrimers can be applied as a non-viral gene delivery platform. In particular, PAMAM and lysine-based dendrimers have shown affinity for DNA molecules inducing the formation of dendriplexes (i.e., complex obtained after the electrostatic interaction between dendrimers and nucleic acids). These structures enhance the cellular uptake of DNA via various mechanisms, such as endocytosis. Additionally, after cellular uptake, the dendriplex facilitates the escape of cells, which are often trapped in endosomes, into the cytoplasm [39].
(iv) **Drug delivery systems**: Dendrimers for drug delivery can be used in two different ways: formulation where they are entrapped in a dendrimer using non-covalent interactions, and nanoconstruction where drugs are covalently coupled on dendrimers. Both strategies allow us to increase the solubility, stability, and oral bioavailability of various drugs [40].

## 5. Janus Dendrimers

Janus particles, named after the two-faced Roman god Janus, are used to describe a unique class of particles that have two distinct sides that can be anisotropic in their composition and surface features. Janus particles have received a considerable amount of interest recently as next-generation “smart” nanomaterials. They provide asymmetry and can thus impart drastically different chemical or physical properties and directionality within a single particle [41]. Broken symmetry offers an efficient and distinctive means to target complex self-assembled materials and realize the emergence of properties (presently) inconceivable for homogeneous particles.

Regarding Janus dendrimers, there are two possible types of these dendrimers. The first and more common type is characterized by having different end-group functionalities and distinct branch structures in each part. The second type is characterized by having only different end-group functionalities while maintaining similar branch structures. The most common method to prepare both types of dendrimers involves the reaction of distinct dendrons possessing complementary functions [42]. This approach utilizes a convergent synthesis strategy, where the dendrons are individually synthesized and subsequently connected to each other using various reactions, such as "click chemistry". Alternatively, another approach involves the utilization of multi-functional cores, which enable the execution of two distinct divergent syntheses to generate the two distinct parts of the Janus dendrimer [43,44].

**Figure 4:**
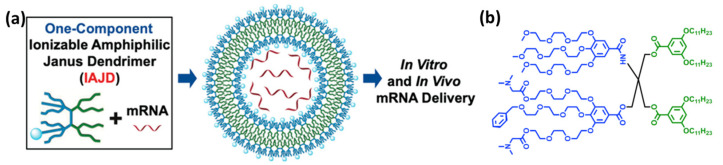
(**a**) Schematic representation of the dendrimersomes based on ionizable amphiphilic Janus dendrimers (IAJD) developed by Percec’s group for the encapsulation of mRNA. (**b**) Chemical structure of a typical IAJD. Reprinted with permission from ref. [46]. Copyright 2021 American Chemical Society.

One of the most interesting properties of Janus dendrimers are their self-assembly capabilities in water when they combine a hydrophilic and another hydrophobic part. Revolutionary research published in 2010 led by Virgil Percec showed that amphiphilic Janus dendrimers can self-assemble in water, forming stable bilayer vesicles referred to as dendrimersomes (Figure 4) [45]. In this work, the author synthesized a library of 107 amphiphilic Janus dendrimers by combining two hydrophobic segments (one aliphatic and one mixed aliphatic-aromatic) and six hydrophilic segments derived from oligoethylene oxide, dimethylolpropionic acid, glycerol, thioglycerol, tert-butylcarbamate, and quaternary ammonium salts. The self-assembly properties of these dendrimers were studied using cryo-TEM, which showed a rich palette of morphologies in water, including vesicles, cubosomes, disks, tubular vesicles, and helical ribbons. Importantly, dendrimersomes can also incorporate pore-forming proteins, coassemble with structure-directing phospholipids and block co-polymers, and offer a molecular periphery suitable for further chemical functionalization.

For the synthesis of the former Janus dendrimers, V. Percec and coworkers developed a synthetic strategy known as the modular-orthogonal methodology [47]. This approach involves using a tetra-functional core, i.e., pentaerythritol, as a core molecule, in conjunction with orthogonal-protecting groups. The protecting groups enable the core to be differentially substituted, ultimately resulting in the formation of a type of Janus dendrimers called twin-twin dendrimers, which usually comprise two identical hydrophobic dendrons and two identical hydrophilic dendrons. Furthermore, this methodology was improved by incorporating tris(hydroxymethyl)aminomethane as the core molecule, enabling the synthesis of dendrimers with three distinct regions [46]. Termed hybrid twin-mix dendrimers, these structures consist of two identical hydrophobic dendrons and two different hydrophilic dendrons (Figure 4b). Percel’s group has successfully exploited these synthetic strategies to obtain a wide range of dendrimers, opening up possibilities for various applications. Some of the notable applications include:

(i) RNA delivery: Efficient delivery of nucleic acids is the key step of genetic nanomedicine. Percec’s group applied the modular-orthogonal methodology to develop a library of 54 amphiphilic Janus dendrimers containing ionizable amines to perform mRNA delivery in vivo (Figure 4) [46]. These Janus dendrimers can encapsulate large quantities of mRNA at an acidic pH (pH 3 to 5), when the amino groups are protonated, due to the electrostatic interactions. In contrast to common lipid nanoparticles that require complex microfluidic technology to encapsulate RNA, these dendrimersomes perform the encapsulation via simple injection, and allow long-term storage.
(ii) Killing of bacteria: Antibiotic resistance is a serious global health problem necessitating new bactericidal approaches such as nanomedicines. Molly M. Stevens’ group has taken advantage of the synthetic strategy developed by Percel’s group for the synthesis of Janus dendrimers to design a dendrimersome-based nanoreactor, with broad-spectrum bactericidal activity [48]. This nanoreactor consists of a dendrimersome with a semipermeable membrane where two enzymes, namely glucose oxidase (GOx) and myeloperoxidase (MPO), are encapsulated. Via external addition of glucose to this system, hypochlorite, which is a highly potent antimicrobial, is produced by the enzymatic cascade. This cascade nanoreactor yielded a potent bactericidal effect against two important multidrug resistant pathogens, Staphylococcus aureus (S. aureus) and Pseudomonas aeruginosa (P. aeruginosa).
(iii) Building mimics of cell membranes: The glycocalyx is the first component of the cell that interacts with the environment, enabling cell communication, cell adhesion, and so on. Importantly, the glycan moieties at the cellular membrane have specific spatial arrangements that can be exploited by some pathogenic bacteria and viruses to attack cells. Studying natural glycocalyx is important to develop synthetic models to dissect structure–function relations. To this end, a type of amphiphilic Janus dendrimers containing carbohydrates in the hydrophilic part has been developed, namely glycodendrimers [47]. The glycodendrimersomes derived from these dendrimers have been shown to mimic some of the supramolecular organization of natural membranes. For example, it was demonstrated that using glycodendrimersomes decorated with mannose, the sugar moieties were organized into periodic nanoarrays without the need for the formation of liquid-ordered phases as assumed necessary for rafts.

A similar example was developed by the group of Guang Yang [49]. They designed a family of dynamic amphiphilic Janus glycopeptide dendrimers containing a β-cyclodextrin as the core and 14 saccharide dendrons as the hydrophilic part. Additionally, they also had seven peptide arms as the hydrophobic region, which were bonded to the core using acylhydrazone bonds. These dendrimers were self-assembled into different glyco-nanostructures with controllable morphologies including glycospheres, worm-like micelles, and fibers. Furthermore, disassembly could be induced using the addition of an acid that hydrolyzes acylhydrazone bonds; thus, they could be applied for the targeting and controllable release of encapsulated guest cargos.

## 6. Supramolecular Dendrimers

Supramolecular dendrimers are a class of highly branched macromolecules formed via the self-assembly of repeating subunits, known as dendrons [50]. Unlike traditional dendrimers, which rely on covalent bonds, these are held together and stabilized using reversible non-covalent interactions, such as hydrogen bonding, electrostatic, π−π, or metal–ligand interactions [51,52]. The dynamic behavior of the physicochemical properties ascribed to non-covalent interactions can be reversible, enabling supramolecular dendrimers to exhibit responsiveness to various external stimuli, such as changes in pH, temperature, and/or the presence of specific molecules [53]. Some of the key properties and advantages of supramolecular dendrimers, in contrast to covalently bonded dendrimers, are as follows: easy modification due to the accessible functional groups on their periphery, and the reversibility and tunability of their dendrimer structure.

While the formation of traditional dendrimers suffers from time-consuming procedures and low yields due to steric congestion, the self-assembly production of supramolecular dendrimers offers considerable advantages: simpler processes, fast and effective creation of the final product, and inherently defect-free assembly [54]. The synthesis of supramolecular dendrimers can be achieved using custom-designed strategies and modifications to tailor the dendritic structures and properties according to specific needs, as discussed previously. One of the most important synthetic steps resides with the selection of the appropriate building blocks, including a dendritic core, peripheral functional groups, and lastly a linker molecule, which will provide the complementary functionalities for self-assembly [54]. Supramolecular dendrimers can be classified based on the type of non-covalent interactions that occur between the dendrimer components into three main classes.

### 6.1. H-Bonding Supramolecular Dendrimers

**Figure 5:**
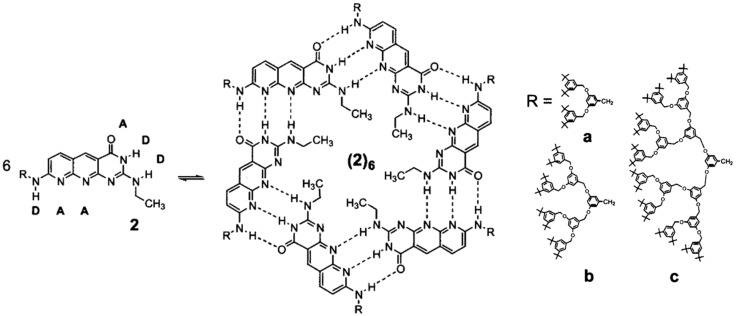
Chemical structure of the monomer and its self-assembly representation to form supra-molecular dendrimers via H-bonding. D, hydrogen bond donor; A, acceptor; R = (a–c). Adapted from ref [56]. Copyright 2002 American Chemical Society.

Utilizing hydrogen-bond-mediated self-assembly represents a potent approach to the construction of extensive structures from small components [50]. Researchers have already described quite well monodisperse supramolecular dendrimers based on hydrogen bonding and their assembly in water [55]. For instance, Zimmerman and co-workers showed not the first but an early example of dendritic structures formed via non-covalent interactions. They constructed these dendritic building blocks based on complementary hydrogen-bonding interactions (Figure 5). The study highlights the dynamic nature of supramolecular assemblies, revealing a self-selected hexamer with alternating dendrons despite numerous potential structures. In this work, steric hindrance plays a crucial role in directing nanoscale assembly, resulting in unique functions from a diverse mixture [56].

On the other hand, a significant advance in the field was introduced by Hirsch et al. The group presented a new approach demonstrating the self-assembly of discrete supramolecular dendrimers, where the motif was no longer based on dendritic subunits [57]. In the reported work, they used a homotropic Hamilton receptor, which typically consists of a binding site or cavity within the dendrimer that can accommodate guest molecules via various non-covalent interactions, such as hydrogen bonding [58]. The Hamilton receptor reported was capable of forming hydrogen bonds with cyanuric and barbituric acid derivatives.

### 6.2. π–π Interactions

**Figure 6:**
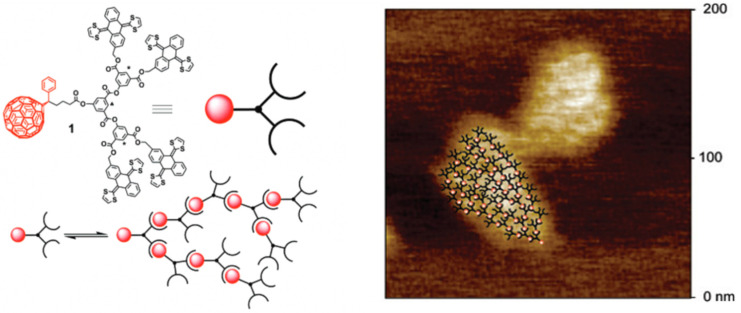
Chemical structure of the monomer (1) and its self-assembly representation to form supramolecular dendrimers via C_60_, along with visualization using AFM. Adapted from ref. [59]. Copyright 2008 American Chemical Society.

Supramolecular dendrimers self-assembled using π–π and van der Waals interactions are less studied than hydrogen bonding. An example of this new type of non-covalent organization of electroactive materials, bridging the gap between dendrimers and supramolecular polymers, was presented by N. Martín [59]. The group of Nazario investigated the potential of a receptor for C_60_ based on the concave–convex complementarity between the curved aromatic surface of the electron donor and the convex exterior of C_60_. They designed a bifurcated molecule (Figure 6) containing two units of their fullerene receptor covalently linked to a PC61BA derivative. The expectation was that the monomer would self-assemble to form treelike supramolecular dendrimers via weak non-covalent interactions, primarily π–π interactions.

### 6.3. Metal–Ligand Coordination

Among the multiple non-covalent interactions, metal–ligand interaction plays a key role due to its high directionality and relatively strong coordinative bond [60]. Balzani and his colleagues pioneered the use of metal branching centers, such as ruthenium and osmium, in conjunction with multidentate ligands to construct metallodendrimer centers [61]. Since then, a significant number of self-assembled dendritic structures have been reported using metal–ligand complexes [62].

### 6.4. Applications of Supramolecular Dendrimers

**Figure 7:**
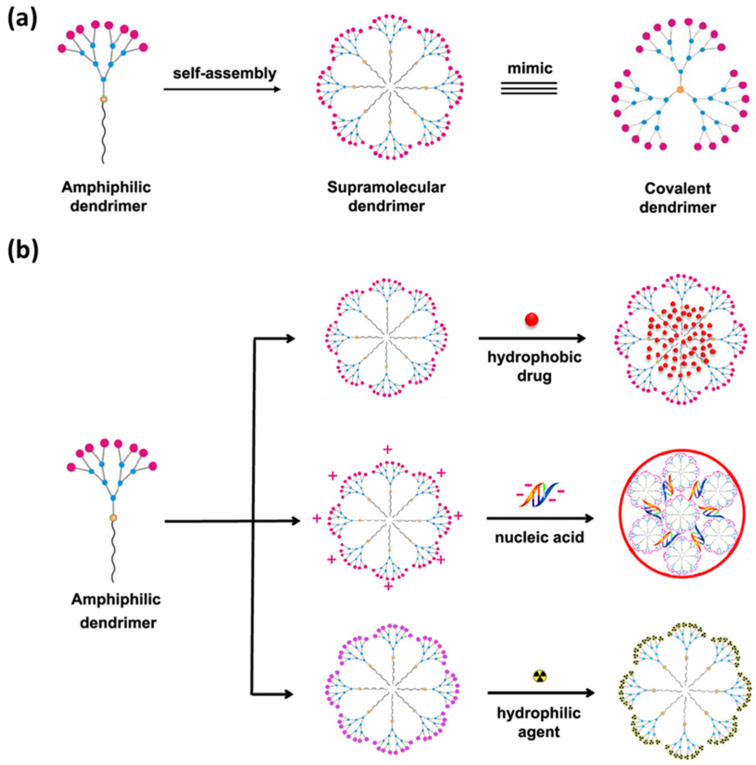
(**a**) Representation of the amphiphilic dendrimer self-assembly as a supramolecular dendrimer that mimics a covalently linked dendrimer. (**b**) Scheme of the delivery of hydrophobic bioagents via supramolecular dendrimers. Adapted from ref. [63]. Copyright 2020 American Chemical Society.

Supramolecular dendrimers are promising candidates for applications in diverse fields, such as drug delivery, diagnostics, materials science, and nanotechnology. Their tunability, reversibility, and stimulus-responsive behavior offer opportunities for designing advanced materials especially for biomedical applications (Figure 7) [63].

(i) **Bioimaging**: Single-photon emission computed tomography (SPECT) is an essential tool in medical imaging due to its unique capabilities, providing crucial three-dimensional information about the functional processes within the body, rather than just structural details. While PET offers higher sensitivity and resolution, SPECT is more readily accessible and less expensive for routine use. Therefore, this essential bioimaging technique was already explored in vivo using supramolecular nanosystems by Peng and his group [64]. The work discusses the development of supramolecular nanosystems based on self-assembled amphiphilic dendrimers with multiple In^3+^ radionuclides at their terminals, serving as SPECT reporters for bioimaging.
(ii) **Stimulus-response delivery systems**: As was already said, one of the most interesting properties of this dendrimer is its dynamic nature. Several groups have taken advantage of this nature to develop stimulus-responsive delivery systems [65]. For example, Feng and colleagues designed a precise in vivo oral protein delivery therapy [66]. The methodology employed by the authors focused on synthesizing benzoboroxole-containing multi-armed poly(ethylene glycol) amphiphilic dendrimers that are sensitive to both pH and glucose levels. The multi-branched structure self-assembled into a supramolecular dendrimer in acidic aqueous solution and exhibited good encapsulation of insulin. However, this dendrimer was quickly disassembled at a neutral pH or in the presence of glucose, releasing the insulin.
(iii) **Nucleic acid delivery**: Safe and efficient nucleic acid delivery still constitutes the major obstacle for clinical implementation. In this regard, Peng and coworkers reported an ionizable supramolecular dendrimer vector, formed via the self-assembly of a small amphiphilic dendrimer, as an effective small interfering RNA delivery system with a favorable safety profile [67].

## 7. Shape-Persistent Dendrimers

Traditional dendrimers incorporate sp^3^ atoms within their framework, conferring a notable degree of structural flexibility. On the contrary, dendrimers comprising solely sp^2^ or sp atoms exhibit a unique architectural rigidity, earning them the designation of "shape-persistent dendrimers". Unlike their traditional counterparts, these shape-persistent dendrimers display an array of distinct characteristics [68]:

(i) **Rigid structure**: They feature a predetermined and rigid structural framework, characterized by branches that are immobilized in precise and spatially defined arrangements.
(ii) **Invariant topology**: Across successive generations, these dendrimers maintain an invariant core architecture and branching topology. Due to this property, it is even possible to prepare 2D shape-persistent dendrimers.
(iii) **Well-defined cavities**: The inherent rigidity of their design imparts substantial mechanical stability, giving rise to well-defined cavities or void regions within the dendritic architecture.
(iv) **High electron mobility**: A striking characteristic of these dendrimers resides in their pronounced propensity for high electron mobility due to their π-conjugated nature.
(v) **Low solubility**: Their solubility is typically low, but it can be increased using the incorporation of solubilizing groups into end-group functionalities.

**Figure 8:**
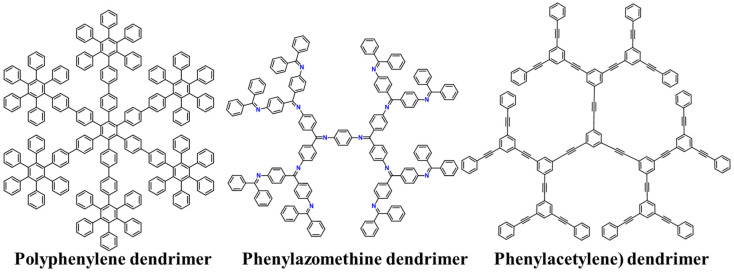
Chemical structures of the main types of shape-persistent dendrimers.

To date, there are three main types of shape-persistent dendrimers (Figure 8).

### 7.1. Polyphenylene Dendrimers

These dendrimers exhibit a tree-like molecular architecture, characterized by an exclusive repetition of phenylene units [69]. Consequently, the structural branches exclusively arise from sp^2^-hybridized carbon atoms, imparting an elevated level of both chemical and thermal robustness. This variant represents an extensively researched category within the realm of shape-persistent dendrimers. Their preparation can be achieved using Suzuki–Miyaura coupling, which involves the use of halogens and boronic acid derivatives [70]. However, this synthetic method leads to the formation of by-products at each step of the synthesis. Consequently, this reduction in the overall yield and the accumulation of by-products can limit the potential for synthesizing higher generations of dendrimers. Another strategy developed by the Müllen group applies Diels–Alder cycloadditions and uses triisopropylsilane (TIPS) as a protection method for the alkynes that do not participate in the reaction [71]. This method considerably reduces the formation of by-products, even when the generation of dendrimers is high. As a result, the preparation of larger dendrimers becomes feasible, with a precisely controlled structure, enhancing the efficiency and success of the synthesis process. Importantly, due to their aromatic structure, polyphenylene dendrimers can exhibit optical properties such as fluorescence and light absorption [72].

### 7.2. Phenylazomethine Dendrimers

In 2000, Yamamoto et al. reported an innovative synthesis of a highly branched, rigid dendrimer with a π-conjugated backbone [73]. The unique feature of this dendrimer lies in the incorporation of imine groups into its structure. This integration leads to a reduction in the intermolecular stacking, which increases solubility. The synthesis of these dendrimers involved a convergent method where the imine is formed using ketone dehydration with aromatic amines, wherein titanium tetrachloride served as an efficient dehydration agent [7]. Using this methodology, the G4 dendrimer was obtained, but it is important to note that as the generation increased, there was a significant reduction in the yield. The first generation yielded 91%, while the last generation only reached 31%.

### 7.3. Phenylacetylene Dendrimers

In 1993, Moore published the synthesis of rigid dendrimers using a convergent approach utilizing phenylacetylene groups [74]. This method gained significance in supramolecular chemistry because of these dendrimers’ ability to form macrostructures via intermolecular stacking [75]. This stacking phenomenon was made possible by the planar structure resulting from the use of these precursors. These dendrimers were synthesized by employing a CuI-catalyzed coupling reaction between 3,5-dibromophenylacetylene and a protected alkyne with a trimethylsilyl (TMS) group. As the generation of the dendrimer increased, a significant rise in the formation of by-products was observed. To address this issue, the reactivity was enhanced by incorporating iodide monomers. These iodide monomers not only facilitated the growth of the macromolecule but also led to a reduction in the formation of undesired by-products.

### 7.4. Applications of Shape-Persistent Dendrimers

Due to their unique and defining attributes, shape-persistent dendrimers have proven to be exceptionally versatile across a wide spectrum of applications. Notably, these dendrimers have garnered substantial attention, giving rise to a large body of literature focused on their multifarious applications. In the subsequent section, we provide a concise overview of these applications [7,69,76]:

(i) **Multichromophore systems**: The aggregation of chromophores in the solid state often leads to red-shifted spectra or even emission quenching. Polyphenylene dendrimers have been shown to be very useful to avoid this aggregation by containing the active functionality in the core. Additionally, they have been also applied to develop multichromophore systems via the incorporation of a large number of chromophores into their branches [76]. In this way, chromophore aggregation is avoided and neighboring chromophores are decoupled from each other.
(ii) **DNA recognition units**: End-group functionalities can be included in these dendrimers to affect their solubility, affinity to specific surfaces, self-assembly behavior, and even electronic properties. For instance, Müllen’s group has applied this strategy to obtain water-soluble core–shell star nanoparticles. By including amino groups in the terminal position of polyphenylene dendrimers, they were able to endow this dendrimer with water solubility. Additionally, they found that these nanoparticles have a strong interaction with DNA, forming complexes with DNA fragments and plasmid DNA even at a sub-nanomolar concentration [77].
(iii) **Guest encapsulation**: The existence of well-defined voids in these dendrimers has been applied to perform encapsulation and even the detection of molecules. This encapsulation is particularly efficient in water due to the high hydrophobicity of the voids. For example, it has been reported the encapsulation of the cyanine dye pinacyanol into a water-soluble polyphenylene dendrimer containing carboxylic acids as terminal functional groups. Furthermore, the internal part of these dendrimers can also be tuned during their synthesis to improve guest recognition. For example, a polyphenylene dendrimer bearing 56 pyridyl functions within its interior showed a surprisingly high affinity and specificity toward the explosive triacetone triperoxide, enabling its detection using quartz microbalances [78].
(iv) **Catalysis**: Phenylazomethine dendrimers contain a large number of imine groups in their chemical structures, and these groups are known for their strong interaction with metal cations [79]. Yamamoto’s group took advantage of these properties to prepare ultrasmall particle sizes (<1 nm) of copper oxide in the interior of these dendrimers. These encapsulated ultrasmall particles were applied as a catalyst, showing excellent catalytic activity in the aerobic oxidation of the CH_3_ group bonded with aromatic rings [80].

## 8. Rotaxane Dendrimers

Rotaxane dendrimers (RDs) represent a fascinating category of molecular structures that integrate the distinctive features of rotaxanes, i.e., intricate molecules comprising interlocked rings threaded onto axles, and dendrimers. This convergence of molecular designs endows RDs with a synergistic combination of the attributes from both parent structures [81,82]. Primarily, this fusion aims to confer dendrimers with the stimulus-responsive features observed in rotaxanes, thereby making them promising platforms for the construction of supramolecular dynamic materials.

**Figure 9:**
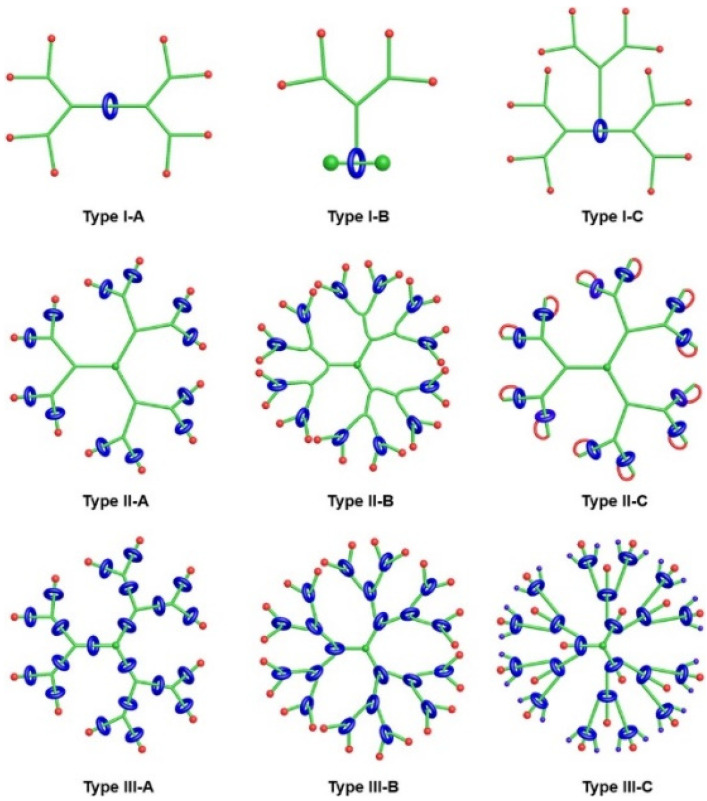
Cartoon representations of the three types of rotaxane dendrimers: type I with rotaxane units as cores; type II with rotaxane units as peripheries; and type III with rotaxane units as branches. The subtypes A, B, and C are named depending where the covalent connection between the dendrons and the rotaxane units are located: the axles (A), wheels (B), or both axles and wheels (C). Adapted from ref. [83]. Copyright 2021 American Chemical Society.

Generally, rotaxane dendrimers can be divided into three types (I, II, and III) based on the positions of the rotaxane units within the dendrimer structures (Figure 9). Additionally, each of these types can be further categorized into subtypes A, B, and C, depending on where the covalent connection between the dendrons and the rotaxane units is located: the axles (A), wheels (B), or both axles and wheels (C) [83].

### 8.1. Type I Rotaxane Dendrimers

**Figure 10:**
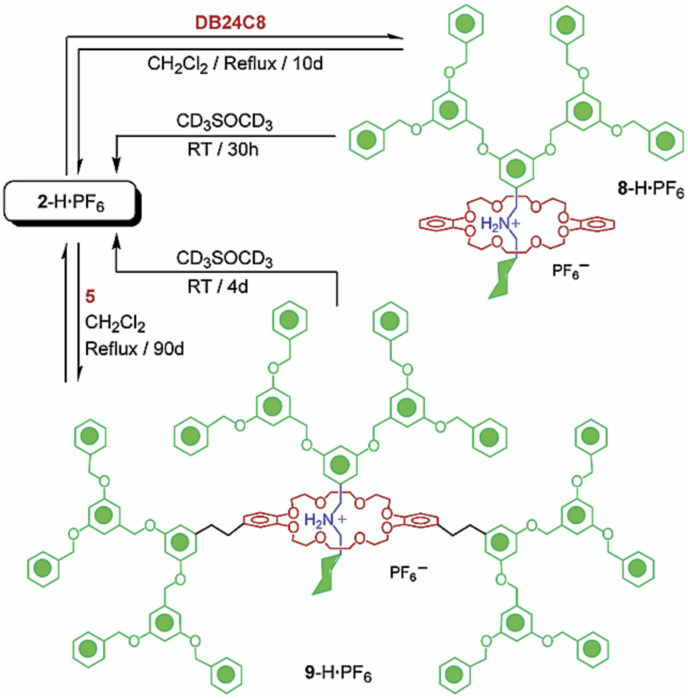
Representation of type I-C rotaxane dendrimer synthesized using the slippage methodology. Reprinted with permission from ref. [86]. Copyright 2002 American Chemical Society.

Stoddart’s research led the forefront in the preparation and exploration of type I dendrimers. This group synthesized several types of I-A RDs following a sequential process involving the threading of the rings onto the axles, followed by a capping step [84]. Additionally, Figure 10 depicts the synthesis of type I-B and type I-C following a slippage methodology that consists of the slippage of a macrocycle over a dumbbell-shaped molecule [85,86]. It is worth highlighting that type-I dendrimers received significant attention during the 1990s and early 2000s. However, the status of this field is relatively inactive, likely attributed to the limited quantity of rotaxanes integrated within these dendrimers, thereby being potentially insufficient to exert a substantial influence on their properties.

### 8.2. Type II Rotaxane Dendrimers

**Figure 11:**
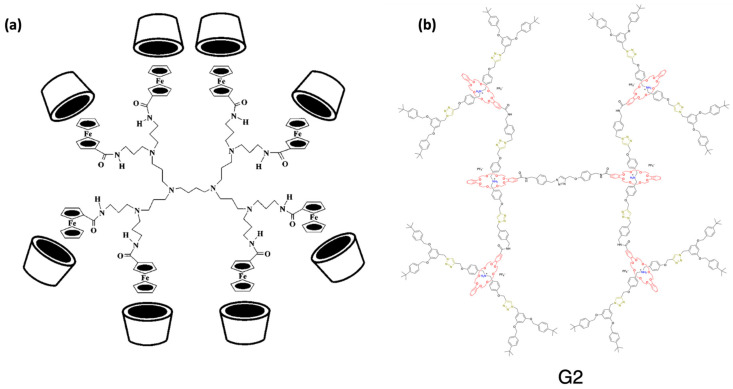
Representations of rotaxane dendrimers: (**a**) structure of a type II rotaxane dendrimer. Here, the dendrimer serves as a three-dimensional template to organize CD receptors in the periphery. (**b**) Type III rotaxane dendrimers for generation 2 (G2). Adapted from ref. [89,91]. Copyright 1997 American Chemical Society and 2018 Springer Nature.

Type II dendrimers refer to dendrimers that possess a periphery adorned with rotaxane structures. However, in contrast to type I, these dendrimers are typically based on pseudorotaxanes instead of rotaxane. A pseudorotaxane is a type of rotaxane that lacks bulky groups at the ends; thereby, it can be dissociated into its two components without breaking the covalent bonds [87]. Like type I RDs, these dendrimers can also be categorized into subtypes A, B, and C, although type II-A is the most abundant class by far. The most typical examples of this type of pseudorotaxane-terminated dendrimer available in the literature exploit the host–guest interactions of cyclodextrins (CDs, Figure 11a) or cucurbiturils (CBs) [88,89]. Remarkably, the presence of CDs in the dendrimer branches enhances their solubility in water [90].

In recent years, several examples of type II-A have been reported that are based on rotaxanes instead of pseudorotaxanes. Other supramolecular hosts different from CB and CD units have been used. For instance, Qu et al. introduced a straightforward synthetic approach via the Co-catalyzed [2+2+2] alkyne cyclotrimerization process that allowed them to transform a [3]rotaxane (i.e., the number in brackets indicates the number of components of the rotaxane) based on a crown ether into a hexa-branched [7]rotaxane in a single step [92]. On the other hand, Goldup and colleagues took advantage of the Cu-mediated azide–alkyne cycloaddition (CuAAC) to synthesize interlocked triazole functionalized porphyrinoids with an excellent yield [93].

### 8.3. Type III Rotaxane Dendrimers

These are dendritic polyrotaxanes whose rotaxane units form part of the dendrimer branches and are also referred to as rotaxane-branched dendrimers. The first example of this type of dendrimer was reported by Stoddart et al. in 1995 [94]. They successfully synthesized a G1, type III-A dendrimer containing [4]rotaxane by means of a slippage procedure. In 2013, Leung et al. described the synthesis and characterization of type III-B rotaxane dendrimers, specifically a G1 [3]rotaxane dendrimer and a G2 [4]rotaxane dendrimer [95].

High-generation type III dendrimers stand out as one of the most interesting types of dendrimers within the domain of dendritic structures. Notably, the augmentation in dendrimer generation yields an exponential increase in the number of rotaxanes within this dendritic type. Consequently, harnessing the stimulus-responsive attributes of rotaxanes enables the conceptualization of sophisticated RDs that exhibit profound alterations in dimensions upon exposure to specific stimuli. For the preparation of these dendrimers, the Leung group published in 2018 the synthesis of a fully organic type of III-B RD of up to G4 generation via a convergent pathway using a CuAAC reaction (Figure 11b) [91]. On the other hand, the H. Yang group developed another strategy to synthesize G4 RDs based on platinum−acetylide moieties, where these moieties served as both bulky stoppers to prevent dethreading and a reactive site that could be used to grow the dendrimers. Using this synthetic approach, they prepared both G4 dendrimers of type III-A and type III-C [83].

### 8.4. Applications of Rotaxane Dendrimers

Rotaxane dendrimers find a wide array of applications, including but not limited to:

(i) **Molecular machines:** Several of the previously mentioned RDs exhibit responsive behavior that can be regulated by external factors such as pH, redox conditions, light exposure, alterations in solvents, and the introduction of anions. This inherent stimulus responsiveness leads to modifications in their dimensions, arising from a coordinated expansion–contraction movement within the integrated rotaxane-branched dendritic structure. Importantly, these structures hold great promise for the development of functional units that enable controlled release of substances in drug delivery systems [96]. Beyond this application, the design of artificial muscles stands out as another exciting prospect, with the potential to transduce chemical energy into mechanical energy [87]. To optimize this distinctive size alteration phenomenon, it is imperative for the dendrimers to harbor the maximum possible count of rotaxanes. Consequently, the most promising contenders for the development of molecular machinery applications are the high-generation type III RDs [96]. This fact was clearly exemplified by a work published by Yang’s group. They described the synthesis of type III-C RDs, including G1, G2, G3, and G4, by employing the platinum–acetylide methodology as previously discussed. These synthesized dendrimers undergo an expansion–contraction dynamic in response to the presence or absence of acetate anions, resulting in a reversible modulation of their sizes within organic solvents. The assessment of these RDs’ swelling ratios using 2-D DOSY experiments revealed the following results: 22.9% for G1, 28.8% for G2, 34.7% for G3, and 38.3% for G4. This illustrated a clear progression as a function of the dendrimer generation [97].
(ii) **Light harvesting:** Photosensitizers play a significant role in light harvesting, which is the process of capturing and converting light energy into another form of energy, such as chemical energy or electrical energy. In this context, rotaxane dendrimers with applications as photosensitizers were designed. The non-covalent modification of dendrimer peripheries offers benefits such as reversibility, selectivity, and adjustability, useful to prevent unwanted interactions between peripheral chromophores, which would cause energy loss during the process [98,99].

## 9. Conclusions and Future Perspectives

Since the first example of a dendrimer was published in the 80s [1], the field of dendrimers has experienced significant advancements, leading to a wide array of available dendrimers today. For example, only considering the classical dendrimers, there is a remarkable diversity in terms of their stability and polarity. Equally, some exhibit exceptional stability, ensuring their longevity and resistance to degradation (e.g., PPI), while others are designed to be biodegradable, allowing for controlled breakdown over time (bis-MPA dendrimers) [27].

Beyond the classical dendrimers, the field has witnessed the emergence of exciting dendrimer architectures that greatly expand the potential applications and capabilities of these nanoscale structures. One remarkable breakthrough in recent years has been the synergistic interaction between molecular self-assembly and dendrimers. This interaction has paved the way for the development of novel types of dendrimers with unprecedented properties. For instance, supramolecular dendrimers have been designed, leveraging the self-assembly of molecular components to form intricate and highly organized structures [54]. Another remarkable advancement is the development of rotaxane-branched dendrimers, which combine the controlled branching of traditional dendrimers with the mechanically interlocked architecture of rotaxanes [96]. Moreover, this synergy has also led to the design of novel self-assembled systems where dendrimers are used as building-blocks, e.g., dendrimersomes [45]. These structures mimic natural cell membranes and hold great potential for applications in drug delivery, nanoreactors, and biomaterials. Certainly, the combination of molecular self-assembly and dendrimers holds tremendous promise for the creation of highly functional materials in the near future.

However, despite the tremendous advance in the dendrimer field, there are still two challenges that limit the application of these materials.

(i) **Synthetic limitations**: The synthetic methodology for traditional dendrimers has undergone significant advancements. For instance, a recent synthetic protocol enables the rapid preparation of G5 dendrimers within a span of less than 12 h [100]. However, a notable challenge remains in achieving high yields and monodispersity in the synthesis of high-generation dendrimers. Divergent synthesis yields high-generation dendrimers with increased yields but compromised branching accuracy. Conversely, the convergent synthesis approach generates monodisperse materials; however, it suffers from diminished yields, and is inadequate for producing high-generation dendrimers due to the steric hindrance between the dendrons. Presently, the most favorable strategies for synthesizing high-generation dendrimers predominantly rely on click chemistry reactions, particularly alkyne–azide cycloaddition, chosen for its notable efficiency [101]. Furthermore, various research groups have endeavored to address the aforementioned challenges by employing solid-phase synthesis as a method for dendrimer preparation. It is important to note that, despite these efforts, this technology is still limited to low-generation dendrimers at the milligram or gram scales [102].
(ii) **Preparation of multifunctional dendrimers**: As was stated in the introduction, dendrimers and proteins share several properties in common. However, a crucial distinction emerges in that while proteins exhibit remarkable asymmetry and highly precise arrangements of functionalities, dendrimers are characterized by remarkable symmetry, often showcasing a singular type of functionality. Although efforts have been made to design multifunctional dendrimers, these materials currently exhibit a random distribution of such functionalities [103]. Undoubtedly, the synthesis of dendrimers incorporating multifunctional arrangements on their surfaces is poised to inaugurate a new realm of highly functional synthetic materials.

A noteworthy development in the realm of dendrimers is the emergence of tecto-dendrimers, often referred to as megamers [104]. Tecto-dendrimers represent a novel approach, where dendrimers serve as reactive modules for constructing nanoarchitectures of greater complexity and larger dimensions. One prominent architectural configuration achieved using this approach involves the assembly of shell-like structures composed of two distinct dendrimer types. To date, these architectures have been mainly applied as contrast agents for the magnetic resonance imaging of tumors [105,106], and as gene delivery platforms [107].

This innovative fusion of advanced synthetic techniques, enabling the creation of multifunctional dendrimers with the concept of tecto-dendrimers, holds immense promise for the future. It opens the door to the development of intricate hierarchical structures with the potential to perform sophisticated functions, perhaps even mirroring biological systems.

## Data Availability

Not applicable.
